# Composition and Diversity of the Culturable Endophytic Community of Six Stress-Tolerant Dessert Plants Grown in Stressful Soil in a Hot Dry Desert Region

**DOI:** 10.3390/jof8030241

**Published:** 2022-02-28

**Authors:** Salam S. AlSharari, Fatma H. Galal, AlaaEddeen M. Seufi

**Affiliations:** 1 Biology Department, College of Science, Jouf University, Sakaka P.O. Box 72341, Saudi Arabia; fatmahgalal@yahoo.com; 2 Department of Entomology, Faculty of Science, Cairo University, Giza P.O. Box 12613, Egypt

**Keywords:** stress-tolerant plants, endophytic fungi, community structure, endophyte-stress correlation

## Abstract

Saudi Arabia is part of a hot dry desert region and is characterized by stressful conditions. The main goal of this research was to identify endophytic fungal (EF) community composition, diversity and abundance in relation to their plant hosts and soil stress. The above-ground parts of six wild plants (*Haloxylon salicornicum*, *Salsola kali, Heliotropium bacciferum, Erica verticillata, Salsola imbricata* and *Bienertia sinuspersici*) were sampled, surface-sterilized and cut into small pieces, which were cultured and incubated for 4–6 weeks. Isolates were grouped and identified by using both morphological and ITS rDNA molecular data. The diversity and community structure of plant-endophyte associations were studied. A total of 455 EF isolates were grouped into 25 different taxa; 21 of which were identified at the species level, 2 at genus level and 2 were unclassified fungi. Here, 95.65% of the identified genera were Ascomycota; of which 36.36, 31.81 and 31.81% were members of the classes Dothideomycetes, Eurotiomycetes and Sordariomycetes, respectively. *S. imbricata* showed the highest isolation rate and colonization frequency (CF%) of EF when compared to other plant species. Additionally, *S. imbricata* demonstrated the highest species richness and species diversity of the EF community predominated by the genus *Fusarium*. Conclusively, the core culturable EF genera of six wild plants were identified (unculturable taxa were not identified in this study). The composition of the EF community was revealed to have a strong correlation to both the electrical conductivity and pH of the soil and a moderate correlation to both the host species and the host family. The abundance and diversity of EF communities of the six plants were environment-dependent.

## 1. Introduction

The microorganisms that inhabit plants without causing any symptomatic diseases to the host plant are called endophytes [1]. Both fungi and bacteria could be endophytic [2]. All host plant tissues, including meristem tissues, are inhabited by microbial endophytes all over the world [2,3]. Especially, endophytic fungi (EF) are believed to associate with plants 400 million years ago [4]. Tissues of various plant species provided a supportive microenvironment for endophytic fungi, and the distribution of EF varies with the host plant. Symbiosis of endophytic fungi–plant has been studied and proved for decades. However, the certain contribution of the host plant to EF biodiversity needs more investigation [5]. The relationship between endophytes and the host plant can be described as a balanced coexistence that ranges from mutual benefit to parasitism [6].

Plant endophytes are regarded to be rich sources of secondary metabolites (SMs) and bioactive compounds (BCs) with commercial and medical uses [7]. Especially EFs produce BCs with varied bioactivities, for example, [7,8]. Production of BCs is, indirectly, triggered by carbohydrates of the host plant [9]. Meanwhile, these compounds increase the resistance of plants to herbivore pests and pathogens [10]. The relationship between the bioactivities of the host and its fungal endophyte needs further investigation, which may shed light on host-endophyte co-evolution and interaction and provide guidance for obtaining bioactive fungal isolates [11].

EF community composition and diversity were reported to depend on soil chemistry [12,13], soil fertility, climatic and environmental conditions of the growing location [14,15], genotype, tissue, age and species of plant host [16,17,18,19,20,21,22,23,24,25,26,27,28,29,30]. Recently, root and soil chemistry has been proven to affect the EF community composition of the host plant [31,32].

Because of their diversity and the huge numbers of fungal endophytic species, which are hosted by a plant species, these symbionts have attracted considerable attention from researchers, for example, in [2,12,14]. Fungal species on our planet were estimated to be composed of 1.5 million species and updated to range from 2.2 to 3.8 [16]. Furthermore, more than one million species of undiscovered endophytic fungi were expected to be described. This estimate was based on the ratio of one vascular plant to four or five fungal species [17]. Studies on the biodiversity, distribution and characterization of endophytes have a significant value in plant sciences. These studies were very helpful to understand and to improve the fitness of plants [18]. Such studies have attracted and still attract the attention of several researchers over decades. Examples of plant endophyte identification using morphological and molecular methods are previously described, for example, in [19]. Consequently, studies that aim to isolate and identify EFs are crucial for any further studies on characterization, diversity, population dynamics of EF, on plant-endophytic fungi relationship, or on screening of EF bioactive secondary metabolites [31,33].

In optimal conditions, each plant species has environmental and nutritional needs that lead to the best productivity and plant health. Some plants are grown in stressful environments exhibiting drought, temperature extremes, mineral deficiency, salinity, etc. Over time, these stresses may lead to the development of stress-tolerant plants which could establish new stress-related responses and thereby keep up continuous growth without suffering stress-related damages. These plants can copy with stresses by a wide range of mechanisms [34]. Understanding stress-related responses is crucial to the successful breeding of stress-tolerant cultivars. Many stress-tolerant plants are endemic in Saudi Arabia. This study selected six endemic wild plants grown in harsh conditions as representative of other wild stress-tolerant plants in the Aljouf region.

Therefore, the main question of this study was to investigate the relationship between endophytic fungal community structure and plant hosts namely: *Haloxylon salicornicum* (Family: Amaranthaceae), *Salsola kali* (Family: Amaranthaceae), *Heliotropium bacciferum* (Family: Boraginaceae), *Erica verticillata* (Family: Ericaceae), *Salsola imbricata* (Family: Amaranthaceae) and *Bienertia sinuspersici* (Family: Amaranthaceae). The relationships between EF communities and the soil chemical properties, abundance and diversity of the EF genera were also investigated.

## 2. Materials and Methods

### 2.1. Sampling and Sampling Site

**Figure 1:**
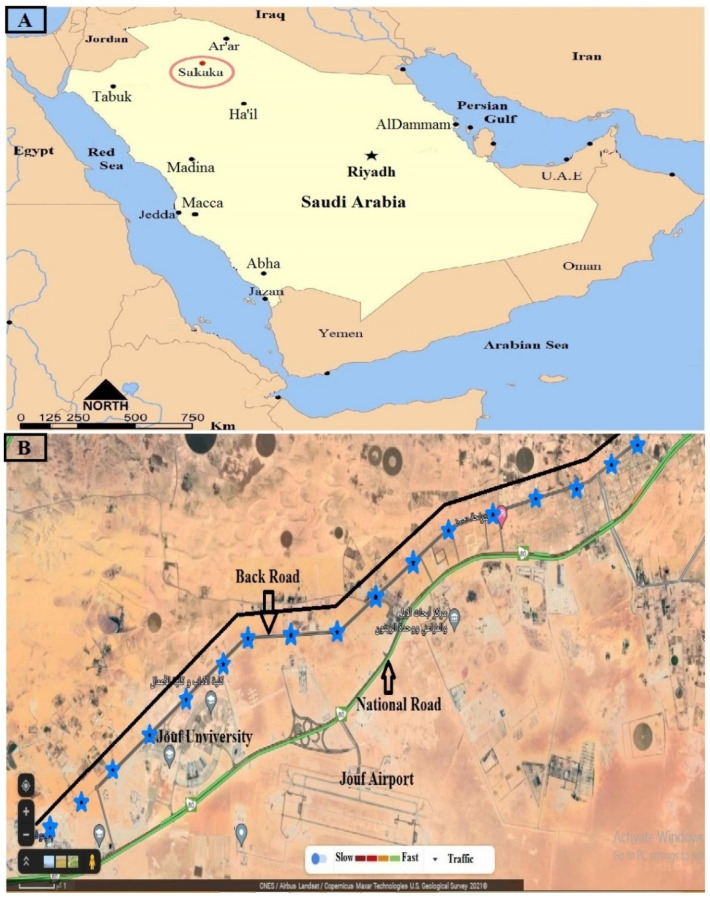
(**A**). Illustrated map of Saudi provinces showing the geographical location of Sakaka city in the Aljouf region. (**B**). Topographic Google Map showing the collection points of samplesalong the back road of Jouf University in Sakaka. Blue asterisks refer to the points of collection.

**Figure 2:**
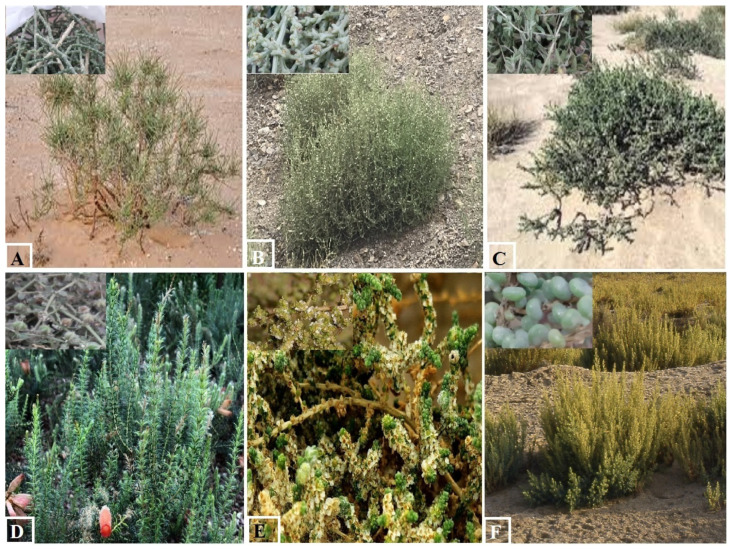
The six wild plants of the present study. (**A**). *Haloxylon salicornicum*, (**B**). *Salsola kali*, (**C**). *Heliotropium bacciferum*, (**D**). *Erica verticillata*, (**E**). *Salsola imbricata* and (**F**). *Bienertia sinuspersici*.

This study was conducted at the back road of Sakaka city, Aljouf Emarah, which is located in the northwest of the Kingdom of Saudi Arabia and serves as the northern entrance of the Kingdom. The altitude of the study site is about 566 m above sea level and is located at the coordinates 29°58′11″ N, 40°12′0″ E (Figure 1). Many wild plants were grown on both sides of the road during the rainy season and this encourages grazing activities on these plants. This road is a part of the hot desert, which is characterized by a stressful arid climate. Samples of six plant species were collected during the first third of January, 2020. These plant species were selected to represent the wild desert plants in the Aljouf region (Figure 2). In addition, they are breeding, persistently, all over the year in a habitat, which is characterized by extremely harsh conditions of temperature, salinity and drought. In addition to the ecological importance of these endemic plant species, they are consumed by pasture animals and have multiple medicinal uses. Annual mean climatic measures were 22.2 °C (min: 4.58, max: 39.75), 30.9% RH (min: 15, max: 57) and light: dark photocycle was 10 L: 14 D (min: 10–11 L: max: 13–14 D). Ten apparently healthy, symptomless individual samples were randomly collected for each plant species. Samples of the same species were collected more than 2 km away from each other. The whole plant was collected and immediately put in a labeled and sterilized plastic bag. Samples were kept at 4 °C until processing within 1 week. Plant species were confirmed by comparison with a reference collection. In addition, the illustrated atlas “Flora of The Kingdom Saudi Arabia Illustrated Series” was used for identification [35].

Multiple rhizosphere soil samples were collected at a depth of 20–25 cm close to each plant sample using manual excavators and drills. Soils were kept in plastic bags and physico-chemical measurements were determined. The study area and points of sample collection were illustrated (Figure 1). A total of 60 samples of six plants were collected from 17 points on the back road of Sakaka city (Figure 1 and Figure 2). Neither protected nor endangered species are applicable for this study.

### 2.2. Surface Sterilization of Plant Samples

In order to remove debris and soil particles, plant samples were thoroughly washed with running water. For minimization of microbial contamination, plants were washed with double distilled water. Surface sterilization was carried out and validated according to Schulz et al. [36]. In brief, plant tissues were immersed in 70% ethanol for 1–3 min, in 4% sodium hypochlorite solution for 3–5 min, washed again with 70% ethanol for 5–10 s, and rinsed three times in sterile distilled water. Plant tissues were finally dried by using sterile paper towels. Surface sterilization was validated by centrifugation of the last rinsing water at 5000× *g* for 10 min. About 500 μL of the supernatant were left, vortexed in the centrifuge tube and a suitable volume was plated onto a PDA medium containing chloramphenicol (200 mg/ L) to prevent bacterial growth. In parallel, small pieces of the surface-sterilized plants were cultured onto the same medium. Both plates were incubated at room temperature and then tested for mycelial growth. No fungal growth in the culture of rinsing water indicated the success of surface sterilization.

### 2.3. Isolation of Endophytic Fungi

The sampling method was planned to isolate as many endophytes as we could. The aboveground part of each sample of the same species was cut into small pieces using a flame-sterilized scalpel (5 mm long). Ten pieces of each sample were randomly chosen for isolation. A total of 600 pieces (6 plant hosts* 10 individuals* 10 pieces) were utilized in this work. Ten pieces of the same sample were plated onto a Petri dish containing PDA medium and chloramphenicol (10 dishes/plant species). To prevent environmental contamination, all steps of isolation were done in a laminar flow hood. Additionally, the scalpel was sterilized for each piece to prevent cross-contamination. Plates were tightly sealed with parafilm, incubated at 28 ± 2 °C for 4–6 weeks. Plates were observed daily for fungal growth. To avoid masking of other fungi, all observed fungal growths were sub-cultured for purification. Active pure colonies were enumerated and transferred to new PDA slants for further studies or kept in cryovials on PDA and 15% glycerol (*v*/*v*) at −80 °C in an ultra-low freezer (New Brunswick, Eppendorf, Ocala, FL, USA). Moreover, negative controls (sealed plates without plant pieces kept inside the laminar flow hood) were used to ensure effective sterilization.

### 2.4. Morphological and Microscopic Identification of Endophytic Fungi

Fungal isolates were identified, morphologically, using macroscopic colony morphology, spore production, spore and hyphal characteristics and asexual reproductive structures. Isolates were, separately, plated onto PDA medium and kept at 28 °C for 7 days. Slides of each fungus were prepared using lactophenol cotton blue stain procedure and examined under a light microscope. All isolates were mainly identified according to the available identification keys [37,38,39,40,41,42].

### 2.5. Molecular Identification of Endophytic Fungi

#### 2.5.1. DNA Extraction

Then, 250-mL sterilized conical flasks containing PD broth were inoculated with each fungal isolate (three replicates/isolate). All flasks were incubated at 28 ± 2 °C for a week and about 100 mg of mycelial biomass was gathered to be processed for DNA extraction. The total DNA of each isolate was purified using the Biospin Fungus Genomic DNA Extraction Kit (Bioer Technology Co. Ltd., Hangzhou, China). The manufacturer’s protocol was followed. Purified DNAs were stored in new tubes at –20 °C until further processing. The concentration, purity and integrity of DNA were assessed as previously described [43].

#### 2.5.2. Oligonucleotides and PCR Amplification

The primer pair ITS1: 5′-TCCGTAGGTGAACCTGCGG-3′ and ITS4: 5′-TCCTCCGCTTATTGATATGC-3′ were designed to amplify approximately 600 bp fragment of highly variable sequences framing the 5.8S-coding sequence and situated between the Small SubUnit- (SSU) and the Large SubUnit- (LSU) coding sequences of the ribosomal operon [43]. Primers were synthesized by MWG-Biotech, Germany. A master mix was prepared in a biosafety cabinet for all PCR reactions (2.5 μL PCR buffer, 1.5 mM MgCl2, 200 μM dNTPs, 1 U Taq DNA polymerase (AmpliTaq, Perkin-Elmer)). A 0.2 mL PCR tube contains 7.5 µL of the master mix, 4 µL of template DNA (≈40 ng), 2.5 µL of 10 pmol forward primer (ITS1), 2.5 µL of 10 pmol reverse primer (ITS4) and 8.5 µL distilled water to reach a final reaction volume of 25 µL. A PCR tube without template DNA was used as the negative control. ABI GeneAmp 9700 thermocycler (Applied Biosystems, Waltham, MA, USA) was used for PCR amplification. The PCR machine was programmed for one cycle of initial denaturation at 94 °C for 5 min; 40 regular cycles include 1 min denaturation step at 94 °C, 1 min annealing step at 55 °C, 1 min extension step at 72 °C. A final extension step for 10 min at 72 °C was performed and then the reaction products were held at 4 °C. PCR products were visualized by 1.5% agarose gel and photographed by a gel documentation system. A 1 Kb ladder (MBI, Fermentas, Waltham, MA, USA) was used to determine the molecular size of the products. On a UV-transilluminator, the desired DNA bands were carefully cut with the least gel fraction. Consequently, pure DNAs were obtained using the QIAGEN gel extraction kit (QIAGEN, GmbH, Hilden, Germany) as recommended by the manufacturer’s protocol.

#### 2.5.3. DNA Sequencing and Sequence Analysis of Fungal ITS rRNA Gene

Two positive PCR products were sequenced bi-directionally for each isolate (to exclude PCR errors) using ITS1 and ITS4 primers, respectively. Sequencing was carried out using the Big Dye terminator sequencing kit (Version 3.1, Applied Biosystems, Waltham, MA, USA) and ABI PRISMTM 3100 DNA sequencer (Applied Biosystems, Waltham, MA, USA).

The resulting sequences for each fungal isolate were reviewed, edited and submitted to BLAST search for appointing putative identities with similar published sequences using the NCBI database http://blast.ncbi.nlm.nih.gov/Blast.cgi (accessed on 15 December 2021). The 23 assured sequences have been deposited in GenBank under the accession numbers from MZ636612 to MZ636634 (Table S1).

### 2.6. Isolation Rate and Colonization Frequency Percentage of EF

The diversity of fungi was studied using the following statistical formulae: Isolation rate (IR) was determined as The No. of isolates obtained from plant segments/The total No. of segments incubated [44]. Colonization frequency percentage (CF%) of the endophytic fungi were calculated as CF% = No. of segments colonized by an endophyte/Total No. of segments analyzed* 100 [45].

### 2.7. Fungal Diversity Indices

Species richness (*R*) was calculated as “the number of different species in a specific community”; Shannon’s diversity index (*H′*) was calculated by Σ (pi ln pi), where pi = n/N; Species evenness (*E*) was also evaluated by H′/ln S [46]; Simpson’s index of dominance (*D′*) was calculated as *D′* = Σ (n/N)2, where n = the total number of isolates of a particular species, while N = the total number of isolates of all species; Simpson’s diversity index = 1 – *D′* [47]; and Berger Parker Dominance Index (*pimax*) was calculated as “the number of individuals in the most dominant taxon relative to the total number of individuals” [48]. Multiple diversity indices were used to emphasize the obtained results using different methods. All the above-mentioned parameters of the six wild plants were calculated using many online webpage calculators (e.g., http://www.alyoung.com/labs/biodiversity_calculator.html). It was last accessed on 10 January 2022.

### 2.8. Statistical Analyses

Soil measurements, IR and CF% experiments were performed in triplicate, and the experiment was repeated three times. Means and standard deviations were computed. A homogeneity test was used to ensure that the means of replicates of the same experiment did not differ significantly. Means were compared by One-Way Analysis of Variance (ANOVA) and Least Significant Difference (LSD). In addition, Two-Way ANOVA was applied to soil properties (electrical conductivity (EC) and pH). Significance was computed at *p* < *0.05*. Spearman’s correlation coefficients (*r*) were calculated at the significance level of *p* < *0.01*. Additionally, principal coordinate analyses (PCoA) were performed to study relationships of EF community structure with the plant host, host family, CF% and soil factors of the six plant hosts. Statistical analyses were carried out using SPSS ver. 24.0 software (IBM Corp., Armonk, NY, USA).

## 3. Results

### 3.1. Climatic and Soil Analyses of the Study Area

The landscape of the collection sites demonstrated very poor vegetation. Analyses of the 10 soil samples revealed sandy and loose, yellow to brownish soil with a very low mean concentration of organic matter (0.99 %). Mean soil composition was demonstrated in Figure S1A. Physico-chemical characteristics of the soil revealed a mean cation-exchange capacity of ≈8.02 cmol/kg. Mean concentrations of macro- and micro-nutrient salts of the soil were also determined (Figure S1B). Temperature (≈–7 to 47 °C), rainfall (annual average ≈ 4.7 mm) and RH% are illustrated in Figure S2A,B.

Additionally, both EC and pH values of the soil samples corresponding to each plant species were statistically analyzed. To reveal if a plant species is stressed by soil, One-Way ANOVA was applied and overall significant differences were observed in both EC (*df =* 5, *F =* 17.99, *p =* 0.00) and pH (*df =* 5, *F =* 13.24, *p =* 0.00).

Interestingly, no significant differences (*p* > 0.05) were observed between soils of the four members of the family Amaranthaceae in both EC (3.24–3.30) and pH (7.92–8.0). The differences between soils of all members of Amaranthaceae were significant (*p* < 0.05) as compared to Boraginaceae and Ericaceae for both EC and pH (Table 1).

**Figure 3:**
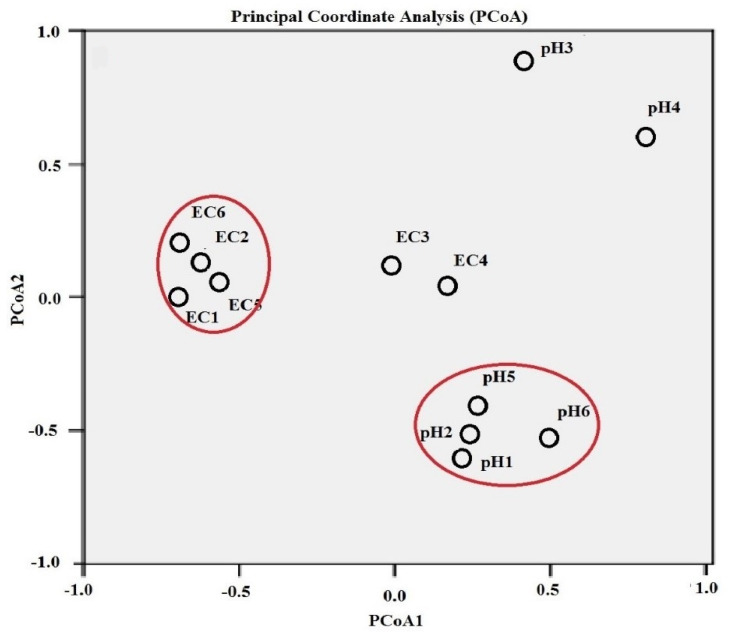
Principal coordinate analysis (PCoA) plot of the EC and pH value of the soil corresponding to the plant host. Both letters of EC1, EC2, EC3, EC4, EC5, EC6 and pH1, pH2, pH3, pH4, pH5, pH6 refer to electrical conductivities (ECs) and pHs of plant hosts as ordered in Table 1. PCoA was done based on a distance-dissimilarity matrix using Euclidean distance to have a plot in the common two-dimensional space showing the ordination of ECs and pH data sets. Red circles show the coordinated soils of Amaranthaceae close to one another in both EC and pH.

Furthermore, PCoA analyses of both EC and pH revealed that soils of all members of Amaranthaceae were coordinated close to one another in both EC and pH (Figure 3).

### 3.2. Identification and Taxonomic Analyses of Fungal Endophytes of the Six Plant Hosts

On the basis of cultural and morphological features, the 455 endophytic fungal isolates were grouped into 25 fungal taxa; 23 of the 25 EF fungal taxa were identified by combining molecular and morphological data. Two fungal taxa were designated as unclassified fungi. Representative culture growths and spore morphology of the identified endophytic fungi are shown in Figure S3.

The taxonomical analysis of the EF-identified fungal taxa is shown (Table 2). Out of 25 fungal taxa, 23 (92.0%) were successfully identified at the genus level. Out of the 23 identified genera, 22 (95.65%) belong to Ascomycota and one belongs to Mucoromycota. Among the 22 Ascomycete genera, 8 (36.36%), 7 (31.81%) and 7 (31.81%) are members of the families, Trichocomaceae and Nectriaceae, respectively. Only one identified Mucoromycete belongs to the family Mucoraceae. Conclusively, 5 *Fusarium* spp., 5 *Aspergillus* spp., 3 *Alternaria* spp., 2 *Penicillium* spp., 2 *Bipolaris* spp., one species of each of the following genera: *Myrothecium*, *Nectria*, *Curvularia*, *Drechslera*, *Dendryphiella* and *Actinomucor* were successfully identified. Two fungal taxa were designated as unclassified fungi (Table 2). Combining morphological and sequence data was very useful for the identification at species level whenever sequence similarity was lower than 99%. In accordance with our results, morphological methods alone are incapable of assigning each genus to its species, while merging morphological with molecular information succeeded in assigning 21 out of 23 genera to their species.

Out of the 23 species, 21 (91.3%) exhibited similarity higher than 97% by BLAST search with related published ITS sequences. Meanwhile, only two species (8.7%) had similarities lower than 97% with related published sequences (Table 2). It is worth mentioning that 13 species (56.5%) out of 23 displayed similarities higher than 99% when compared with the corresponding published sequences (Table 2).

### 3.3. Effect of Plant Host on the EF Community Composition Using PCoA Model

**Figure 4:**
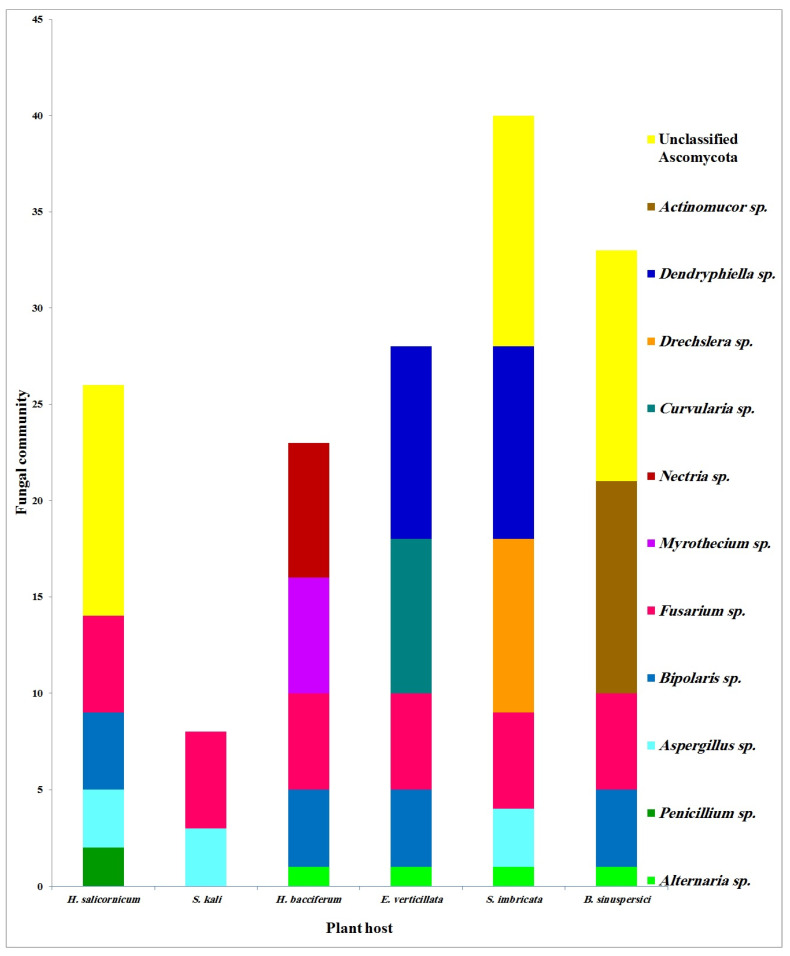
Stacked bar chart showing the common endophytic fungal genera isolated from the six wild stress-tolerant plant hosts.

Figure 4 summarizes the isolation of the common EF genera from their host plants. Out of the six plant species, four species belong to the family Amaranthaceae (*H. salicornicum, S.* *kali, S. imbricata* and *B. sinuspersici*), one species belong to the family Ericaceae (*E. verticillata*) and one species belong to the family Boraginaceae (*H. bacciferum*). All these plant species are growing in extremely stressful conditions (salt, pH, drought and thermal stresses) as previously described (Figures S1 and S2 and Table 1).

Regarding the plant species, five EF genera were isolated from each one of the plant species: *H. bacciferum*, *E. verticillata* and *S. imbricata*; four EF genera were isolated from each one of the plant species: *B. sinuspersici* and *H. salicornicum* and two EF genera were isolated from *S.* *kali*. Thus, out of six plant species, five were inhabited by ≥4 EF genera.

Concerning fungal genera, *Fusarium* was the most frequent genus as it was hosted by all of the six studied plants. Some genera were found to colonize more than one host. Both *Alternaria* and *Bipolaris* colonized four hosts, *Aspergillus* colonized three hosts and *Dendryphiella* colonized two hosts. Meanwhile, other genera colonized a specific host. *Penicillium* colonized *H. salicornicum*, both *Myrothecium* and *Nectria* colonized *H. bacciferum*, *Curvularia* colonized *E. verticillata, Drechslera* colonized *S. imbricata* and *Actinomucor* colonized *B. sinuspersici* (Figure 4). From a different point of view, Ascomycetous genera were recovered from the six studied plants. However, the unique Mucoromycetous genus, *Actinomucor*, was specifically recovered from *B. sinuspersici*. It is clear that the core endophytic genera embraced *Fusarium, Alternaria, Bipolaris, Aspergillus* and *Dendryphiella*. These five genera colonized more than one host species (Figure 4).

**Figure 5:**
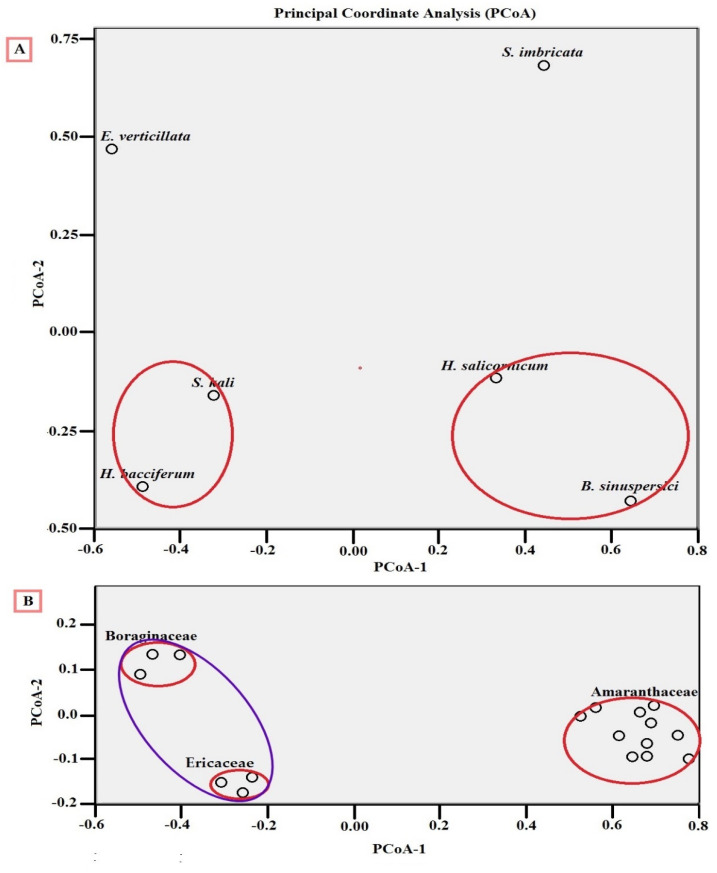
Multidimensional scaling using principal coordinate analysis (PCoA) plots of the community structure of EF in relation to: (**A**) the plant host and (**B**) the family of the plant host. The analyses were conducted based on a distance-dissimilarity matrix using Euclidean distance to have a plot in the common two-dimensional space showing the ordination of plant species and plant family data sets of EF community structure. Red and purple circles show the close coordination of EF communities of the same plant host and plant family.

PCoA model was calculated on the basis of dissimilarity distances between the EF community of the six plant hosts (Figure 5A). Data were Gaussian or normally distributed. The PcoA revealed that both *S. kali* and *H. bacciferum* were standing closer to one another and *H. salicornicum* and *B. sinuspersici* were ordinated closer to one another (Figure 5A). Additionally, the calculated PcoA model of the EF community of the three plant families was carried out (Figure 5B). PCoA revealed that both Boraginaceae and Ericaceae families were ordinated closer to one another. Meanwhile, the EF community of Amaranthaceae stands apart from them (Figure 5B).

Correlation analyses between the EF communities of the six plant species confirmed a positive significant moderate correlation (*r =* 0.648) between *H. salicornicum* and *B. sinuspersici* (Amaranthaceae) by using the two-tailed Spearman’s coefficient at *p >* 0.05 significance level (Table 3A). However, no significant correlation (*r <* 0.5) between families was confirmed by using Spearman’s coefficient (Table 3B).

### 3.4. Effect of Soil EC and pH on the EF Community Structure

Two-way ANOVA revealed significant overall individual effect of both EC (*df* = 5, *F*(5,54) = 17.99, *p* = 0.0, *η_p_*^2^ = 0.625) and pH (*df* = 5, *F*(5,54) = 13.24, *p* = 0.0, *η_p_*^2^ = 0.551) on the EF community structure of the six plants. However, the combined effect of EC and pH was insignificant (*df* = 10, *F*(10,54) = 0.076, *p* = 0.892, *η_p_*^2^ = 0.004).

Additionally, correlation analyses using the two-tailed Spearman’s coefficient revealed strong positive correlations (r > 0.886–0.961) between pairs of the four members of Amaranthaceae which share soil salt-stress (Table 4). Meanwhile, strong positive correlations (r > 0.876–0.980) were shown between the same pairs that shared soil alkalinity-stress (Table 4).

### 3.5. Effect of the Plant Host on Some Abundance Measures in Relation to Endophytic Fungi

A total of 455 fungal isolates were obtained from the 600 plant segments incubated. The highest number of isolates was recovered from *S. imbricata* (100 isolates). Whilst the lowest number of isolates was recovered from *S. kali* (62 isolates). Furthermore, 83, 85, 67 and 80 isolates were recovered from *H. bacciferum, E. verticillata, H. salicornicum* and *B. sinuspersici*, respectively.

The isolation rate of the EF on the six studied plants was investigated (Figure S4). The six plant species demonstrated percentage isolation rates (IR) of more than 50%. The highest and the lowest IRs (number of infected segments) were demonstrated by *S. imbricata* (70%) and *S. kali* (50%). One-way ANOVA elucidated that the overall difference in the isolation rates of EF (total No. of isolates obtained from plant segments/Total No. of segments incubated) was significant (*df* = 5, *F* = 4.753, *p* = 0.001). Post-hoc tests clarified significant differences in the isolation rates of EF between the plant, *H. salicornicum* and both of *S. imbricata* and *B. sinuspersici*; between the plant, *S. kali* and each of *H. bacciferum*, *E. verticillata*, *S. imbricata* and *B. sinuspersici*; and between the plant, *S. imbricata* and *B. sinuspersici* (*p* < 0.05) (Figure S4).

Percentage colonization frequency (CF%) of EF (No. of segments colonized by an endophyte/Total No. of segments analyzed* 100) was studied for the six plant species (Figure S5). CF% of endophytic fungi showed obvious significant overall variation (*df* = 5, *F* = 5.65, *p* = 0.003) in all plant species. The highest number of segments colonized by endophytes (80.2) were reported for *S. imbricata*. On the other hand, *S. kali* exhibited the lowest CF% (50.2) when compared to other plant hosts. Multiple comparison tests demonstrated significant differences in the CF% between *H. salicornicum* and *S. kali* (*p* < 0.05). It is discernable that the number of segments colonized by endophytes (CF%) in the case of *S. imbricata* is significantly higher (*p* < 0.05) when compared to all other plant species (Figure S5).

**Figure 6:**
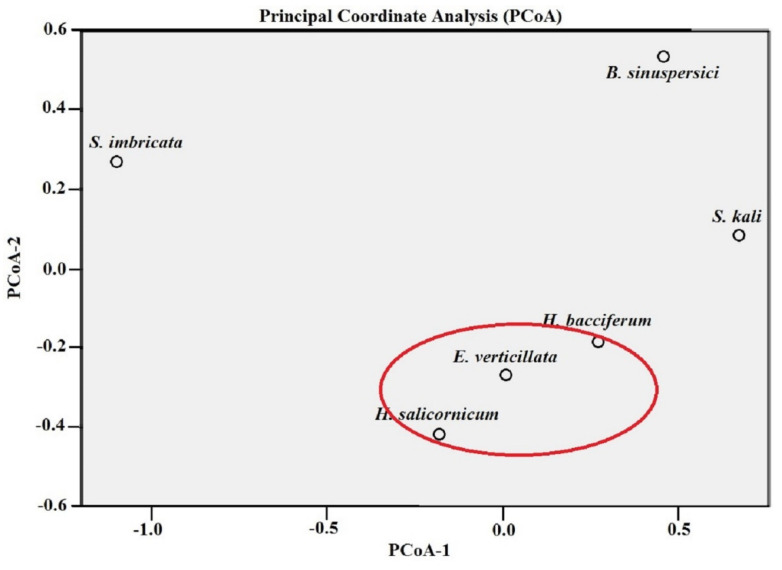
Multidimensional scaling using principal coordinate analysis (PCoA) plots of the CF% of EF of the six plant hosts showing ordination of plant species data sets of CF%. PCoA was done based on a distance-dissimilarity matrix using Euclidean distance of CF% to have a plot in the common two-dimensional space. Red circles show the close coordination of CF% of three correlated plant hosts.

Figure 6 demonstrates the calculated PCoA model of CF% of the six plant hosts. PCoA revealed that *H. bacciferum, H. salicornicum* and *E. verticillata* were ordinated closer to one another (Figure 6). Correlation analyses between the CF% of the six plant species were carried out using Spearman’s coefficient (Table 4). *H. bacciferum, H. salicornicum* and *E. verticillata* demonstrated positive significant strong (*r* > 0.995–1.000) correlations using two-tailed Spearman’s coefficient at *p* > 0.05 significance level. Meanwhile, *S. kali, H. bacciferum* and *E. verticillata* showed positive significant strong (*r* > 0.995–1.000) correlations using Spearman’s coefficient at *p* > 0.05 significance level (Table 4). Conclusively, *S. imbricata* showed the highest abundance measures (IR and CF%) of the EF when compared with the other host plants.

### 3.6. Diversity Indices of the EF Community in Relation to Plant Host

Table 5 highlighted some diversity indices of the EF isolated from the six wild plants. Multiple diversity indices were used to emphasize the obtained results. *S. imbricata* demonstrated the highest species richness (7.0) and the lowest evenness (0.14). Regarding Simpson’s index of diversity, the highest diversity of EF community was realized for *S. imbricata* (least value (0.0476) of Simpson’s diversity index). However, the value of Shannon’s diversity index (0.1) did not realize *S. imbricata* diverse EF community because this index considers both richness and evenness. On the other hand, the higher dominance indices (Simpson’s index = 0.9524 and Berger Parker index = 97.6%) of *S. imbricata* indicated that the EF community is predominated by the most prevalent species “*Fusarium*” (Table 5).

## 4. Discussion

It is crucial to identify species with deeper, faster, more accurate and economically effective methods [49]. However, morphological and culture-dependent methodologies are time-consuming, vigorously robust and stand in need of expert microbiologists to discriminate morphologically comparable species [50]. By using sequence data of ITS rRNA gene, we succeeded to assign isolates up to their genera via RDP Classifier. BLAST search and multiple alignments were very helpful in assigning genera to their most likely species in 19 genera out of 23. Meanwhile, using both morphological and molecular data succeeded in assigning the species of two additional genera (*Bipolaris spicifera* and *Aspergillus flavus*). Two isolates were identified at the genus level (*Myrothecium* and *Drechslera*). In total, 21 isolates out of 23 were identified at the species level and 2 at the genus level. In parallel, molecular methods succeeded to classify and identify several EF species [51,52]. The ITS1 and ITS4 primers were efficiently used in identifying fungi [43]. Positives and negatives of microbial identification by different techniques were studied, intensively [49,53]. Our sequences created identities from 95.92 to 100% when compared to the closest sequences in GenBank. Out of 23 sequences, six created similarities less than 98%. Concerning the general rule of thumb and its updates [54,55,56], only two novel species of the genus *Aspergillus* were reported in our study (*Aspergillus flavus* (95.92%) and *Aspergillus terreus* (96.58%)). The latest update of the 97% rule suggested an optimal threshold of 99.6% identity to indicate new species of filamentous fungi when using the ITS rRNA gene [57]. Pearson [58] recommended the use of E-values and bit scores (bits > 50) in deducing homology [58]. Relying on the aforesaid updates, 21 out of the 23 identified species could be regarded as new species or subspecies. The number of identified genera varied according to the method of isolation and identification. Although the culture-independent sequence-based method identified more genera, some fungi were identified only by culture-dependent isolation methodology [24].

Although the tested plants were asymptomatic, some isolated EF species were well-known pathogenic fungi including some *Fusarium* species, which exhibited high dominance and persistence in this study. This was so disquieting because it was uncertain whether these isolates were latent pathogens or they played another beneficial role. For instance, some species of the core-genera *Fusarium, Alternaria, Bipolaris* and *Aspergillus* were presented as pathogens of numerous plant and cereal species resulting in reduced product quality and quantity [59,60,61,62]. Interestingly, the alignment of some of our EF species with some corresponding previously isolated pathogenic species created very low similarities for the tested sequences (less than 73%). This point is worthy of being investigated, deeply, in future work. Moreover, endophytic species of these genera were reported to promote plant performance in stressful environments through varied mechanisms. For example, treatment with the endophyte *Alternaria alternata* promoted growth and increased drought tolerance in wheat plants. Plants inhabited with the endophyte were effectively overcome free radicals induced by stress [63]; application of the endophyte, *Aspergillus awamori* improved the performance of mung bean seedlings in terms of antioxidant enzymes, biochemical measures, seedling growth, ionic status and indole acetic acid content of the plant under salt stress [64]; inoculation of the endophyte, *Aspergillus* *niger* enhanced thermal stress tolerance of soybean and sunflower through antioxidant and metabolic pathways [65]; administration of the endophyte, *Bipolaris* sp. CSL-1 attenuate the effects of salinity stress on soybean via changing antioxidant status, endo-hormones and expression of stress-related genes [66]; colonization of a salt-susceptible rice variety by the endophytic fungus, *Fusarium* sp. resulted in reducing the number of alternative splicing occurrences under saline stressful conditions [67] and the co-cultivation of tomato seedlings and the endophyte *Fusarium solani* confer protection against the strong detrimental effects of drought under laboratory conditions [68]. Furthermore, the endophyte, *Fusarium oxysporum* could control pathogens via direct or indirect endophyte–pathogen interactions. The direct way includes antagonism, parasitism and competition for food or housing; however, the indirect one involves stimulation of defense pathways of the host [69]. Interestingly, the marine species *Dendryphiella salina* has colonized two salt-tolerant hosts: the halophyte, *S. imbricata* and the disease-resistant, *E. verticillata*. In accordance, the endophytic *Dendryphiella salina* from different locations and climatic zones demonstrated high adaptation to salt stress and pH values between 6.5 and 8.0 [70]. Thus pathogenic fungi may be new emerging species or may become latent pathogens as a result of the environmental stress as shown by the soil alkalinity (pH) and salt (EC) stresses in our study. Also, the EF may have altered the endophyte–host relationship as a result of the co-existence over time. That is, rapport building between EF and their host plants may have been developed via particular fungal–host interchanges that have been recognized as a continuum of antagonism, neutralism and mutualism [15].

The other six identified EF non-core genera were reported to have varied bioactivities. The endophytic *Actinomucor, Penicillium, Nectria, Curvularia,* *Drechslera* and *Myrothecium* were found to promote tolerance of their hosts against various types of stress [71,72,73,74,75,76]. As a whole, the role of the endophytism of EF in stress-tolerant species is worthy of future investigation. From the aforementioned arguments, we concluded that the same fungal species may be pathogenic and as a result of becoming endophyte colonizing a plant host, it transforms from a pathogenic organism into a beneficial fungus. Moreover, it participates in confronting stressful environmental conditions of the host (thermal, water, pH and saline stress) through direct and indirect mechanisms. These alterations have been established due to the co-existence of the endophyte-host over time.

Many factors such as genotype, tissue, age and species of the host, endophytic species and environmental conditions have been reported to involve in EF community composition and diversity within the host plant [25,26,27]. Our results clarified that there were moderate correlations between the genus composition of the EF community and both the species and the family of the host plant. In addition, salt (EC) and alkaline (pH) soils proved to have a significant individual effect and strong positive correlation with the EF community structure. Several genera were isolated from more than one host and more than one plant family (*Fusarium, Alternaria, Bipolaris, Aspergillus* and *Dendryphiella*). That is most probably because the unique influencing factor in our study is the common stressful environment of the study area (thermal, drought, salinity and alkalinity), in which the plants grew. Agreeable results have been demonstrated that variations of EF community were location or ecosystem-dependent, for example, in [24,77]. PCoA model and correlation analysis clarified that the EF communities of both *H. salicornicum* and *B. sinuapersici* showed a strong positive correlation (they shared two out of five EF core genera). In addition, the EF communities of both *S. kali* and *H. bacciferum* shared one EF genus. These results suggested that EF communities of the above-mentioned pairs are more similar. Meanwhile, the PCoA model clarified that the EF communities of both Boraginaceae and Ericaceae families are more similar (they shared three out of five EF core genera). However, the four hosts of the family Amaranthaceae exhibited unique ordination because 7 out of 11 identified EF genera were isolated from one or more members of Amaranthaceae. Integration of these data clarified that EF community structure demonstrated moderate specificity to both the host plant and the family of the host plant. This may be attributed to the fact that several EF genera were shared between more than one host plant and between more than one plant family. However, several wild plant seeds shared only two core microbes, and they had unique endophytic microbiota [78]. Possibly, wild plants acquired their endophytic microbiota from the surrounding soils, and the soil microbiome could enter and be differently distributed within plant tissues, especially for endophytic fungi. Therefore, EF community composition seems to be soil/environment-dependent.

More than 50% of both endophytic fungal IR and CF% were exhibited by all the studied plant hosts. This may be attributed to that they were grown in harsh stressful environmental conditions. Evidence for soil alkalinity and salt stresses were provided in this study. Endophytes conferring salt and heat tolerance were previously reported [79]. Additionally, statistical analyses of our results suggested that the isolation rate (IR) and CF% of EF were partly host-species-related (significant differences in some but not all cases). Moreover, the reason for the highest CF% of *S. imbricata* may be due to its halophytic nature. The halophytic genera like *Salsola, Atriplex* and *Haloxylon* were reported to exhibit 100% CFs in a dry ecosystem [80]. Species diversity and abundance measures of EF communities were found to be tissue and location-dependent in soybean [24]. Integration of our data and those presented in previous studies concluded that the number of EF isolates and species to be identified from a host plant were may depend on host [19,28], location [29,30] and/or methodology [24]. The lower CF% presented by many researchers could be attributed to the antimicrobial SMs produced by the host plant and the inhibited EF colonization [81]. PCoA model and correlation analysis clarified that the CF% of the triplex (*H. bacciferum, H. salicornicum* and *E. verticillata*) are more similar with a positive correlation between their pairs. Meanwhile, the CF% of the two plant species *S. imbricata* and *B. sinuspersici* did not display similarity or correlation. In the light of the highly stressful environment in our study site, the direct relationship (positive correlation) of the CF% of several plants together could be because they share the colonization of the EF species, share the harsh stressful environmental conditions and/or share similar internal microenvironment of the hosts. The genotype, tissue, age and species of the host, endophytic species and environmental conditions have been reported to be involved in powerful endophytic colonization within host plants [25,26]. The EF community structure in a specific plant host was suggested to have a synergistic effect to promote the host growth and stress tolerance. However, some EFs could play an antagonistic role for other pathogenic EF in order to improve host performance.

Multiple diversity indices indicated that *S. imbricata* demonstrated the highest species richness, species diversity of EF community, which is predominated by the genus *Fusarium*. The diversity of the EF community was reported to be higher in wildflowers than in greenhouse-cultivated flowers [82]. Higher diversity was also reported in many habitat-stressed plants [83,84]. Additionally, much evidence has supported the strong link between the EF community and the promotion of plant growth and/or health [85]. Several endophytic fungi could contribute to the host plant growth, health and enhanced its adaptation to stresses either in direct and/or indirect ways [85,86,87]. Consequently, the higher EF diversity may lead to better plant health by increasing the probability of more beneficial fungi in the EF community composition. These ecosystem-specific EF might be soil or environment-originated at first, and consequently colonized the plants grown in the same environment. This is why the core EF genera were found to colonize more than one host of the studied plants, sharing the same soil characteristics, the same climate and growth conditions. It could be suggested that the environment and soil characteristics are the main stimulators of both EF community structure, species abundance and EF diversity [88]. The host-specific genera could be a result of the difference in the internal plant microenvironment or due to the intraspecific interaction of the EF community within the host species. Finally, we believe that the endophyte–host interaction of each one of the core-fungal genera is worthy of being investigated in future work, especially, for economically important and stress-tolerant plant species.

## 5. Conclusions

In short, this study described the structure of EF communities associated with wild stress-tolerant desert plants grown in the Aljouf region of the Kingdom of Saudi Arabia. The core EF genera of six hosts were recognized (*Fusarium, Alternaria, Bipolaris, Aspergillus* and *Dendryphiella*). It was clarified that the genus composition of the EF community was moderately correlated with both the host species and the family of the host plant. Interestingly, EF community composition was significantly affected and strongly correlated with EC (salty) and pH (alkaline) soil stress. The abundance and diversity of EF communities of the six plants were influenced by the stressful environment of the study area. These results would simply provide a conception of the potential endophyte–host interaction mechanisms. In addition, it is a primary step for future deep screening of the potential unculturable EF within these plants, EF bioactivity and for pot cultivation of wild medicinal plants, after deposition of the plant strains in a public collection. Finally, the five core genera are worthy of being explored and their contribution to host stress tolerance in can be explored in future studies.

## Figures and Tables

**Table 1:** Soil electrical conductivity (EC) and pH of the six wild plant hosts. The mean EC and pH of the six wild plant hosts. The lower case letters on the numbers refer to the significance level. Different letters refer to significant differences and the same letters refer to insignificant differences at *p* < 0.05 using LSD post hoc tests.

Plant Species	EC (*n* = 10) Mean ± SD	pH (*n* = 10) Mean ± SD
*H. salicornicum*	3.27 ± 0.23 ^a^	8.00 ± 0.25 ^a^
*S. kali*	3.25 ± 0.19 ^a^	7.92 ± 0.22 ^a^
*H. bacciferum*	2.84 ± 0.13 ^b^	7.53 ± 0.14 ^b^
*E. verticillata*	2.84 ± 0.10 ^b^	7.48 ± 0.12 ^b^
*S. imbricata*	3.28 ± 0.18 ^a^	7.92 ± 0.19 ^a^
*B. sinuspersici*	3.30 ± 0.14 ^a^	7.94 ± 0.25 ^a^

**Table 2:** A key table showing taxonomical analysis and similarity measures (using ITS rRNA gene) of the endophytic fungal morphotypes isolated from the six wild stress-tolerant plant hosts.

Taxonomic Rank					Similarity Measures		
Morphotype	Suggested Genus	Suggested Species	Division	Class	Total Score	Query Cover	Max. % Identity
2 (WF12)	*Alternaria* sp.	*A. infectoria*	Ascomycota	Dothideomycetes	1282	100%	97.86%
9 (WF32)	*Alternaria* sp.	*A. alternata*			1725	99%	98.96%
12 (WF41)	*Alternaria* sp.	*A. botrytis*			2320	99%	99.30%
5 (WF21)	*Bipolaris* sp.	*B. spicifera*			1967	100%	99.54%
21 (WF62)	*Bipolaris* sp.	*B. sorokiniana*			2669	100%	99.66%
14 (WF43)	*Curvularia* sp.	*C. lunata*			1949	100%	98.99%
19 (WF55)	*Dendryphiella* sp.	*D. salina*			2047	100%	99.12%
17 (WF53)	*Drechslera* sp.	*Drechslera* sp.			2078	100%	98.88%
3 (WF13)	*Penicillium* sp.	*P. citrinum*		Eurotiomycetes	870	97%	97.10%
Ag (WF13)	*Penicillium* sp.	*P. griseofulvum*			2333	99%	98.85%
4 (WF14)	*Aspergillus* sp.	*A. terreus*			431	96%	96.58%
7 (WF23)	*Aspergillus* sp.	*A. flavus*			835	99%	95.92%
11 (WF34)	*Aspergillus* sp.	*A. niger*			1860	99%	100%
15 (WF51)	*Aspergillus* sp.	*A. clavatus*			2362	100%	98.65%
Ag (WF34)	*Aspergillus* sp.	*A. awamori*			2457	100%	99.56%
6 (WF22)	*Fusarium* sp.	*F. venenatum*		Sordariomycetes	1303	100%	99.31%
13 (WF42)	*Fusarium* sp.	*F. oxysporum*			1245	100%	97.17%
16 (WF52)	*Fusarium* sp.	*F. proliferatum*			2161	100%	99.17%
18 (WF54)	*Fusarium* sp.	*F. fujikuroi*			1786	100%	99.09%
Ag (WF22)	*Fusarium* sp.	*F. solani*			3085	100%	99.13%
10 (WF33)	*Nectria* sp.	*N. cinnabarina*			2194	100%	99.92%
8 (WF31)	*Myrothecium* sp.	*Myrothecium* sp.			680	99%	97.51%
20 (WF61)	*Actinomucor* sp.	*A. elegans*	Mucoromycota	Mucoromycetes	2348	100%	99.53%
1 (WF11)	Unclassified Fungus	---	Ascomycota	---	---	---	92%
22 (WF63)	Unclassified Fungus	---		---	---	---	92%

**Table 3:** Summarized table of Spearman’s correlation showing: (**A**). Correlated pairs of the six plant hosts in relation to EF community structure. (**B**). Correlated pairs of the three plant families in relation to EF community structure.

(A)		
Plant Species		*R (Spearman)*
***H. salicornicum***	*S. kali*	0.703 *
	*H. bacciferum*	0.121
	*E. verticillata*	0.121
	*S. imbricata*	0.069
	*B. sinuspersici*	0.341
***S. kali***	*H. bacciferum*	0.081
	*E. verticillata*	0.081
	*S. imbricata*	0.412
	*B. sinuspersici*	0.219
***H. bacciferum***	*E. verticillata*	–0.065
	*S. imbricata*	–0.284
	*B. sinuspersici*	0.166
***E. verticillata***	*S. imbricata*	0.324
	*B. sinuspersici*	0.166
***S. imbricata***	*B. sinuspersici*	–0.092
**(B)**		
**Plant Family**		***R (Spearman)***
***Amaranthaceae***	*Boraginaceae*	–0.069
	*Ericaceae*	–0.069
***Boraginaceae***	*Ericaceae*	0.267

* Correlation is significant at the 0.05 level (two-tailed).

**Table 4:** Summarized table of Spearman correlations showing the correlated pairs of the EF community of six plant hosts in relation to the soil electrical conductivity (EC), soil pH and fungal colonization frequency (CF%).

Plant Species		Spearman’s Correlation Coefficient (*R*) of		
		EF Community and Soil EC	EF Community and Soil pH	EF Community and Fungal CF%
***H. salicornicum***	*S. kali*	0.957 **	0.967 **	0.949
	*H. bacciferum*	0.009	–0.079	0.949 **
	*E. verticillata*	0.064	0.061	0.949 **
	*S. imbricata*	0.894 **	0.939 **	–0.316
	*B. sinuspersici*	0.920 **	0.954 **	–0.632
***S. kali***	*H. bacciferum*	0.003	–0.146	1.000 **
	*E. verticillata*	0.098	0.034	1.000 **
	*S. imbricata*	0.954 **	0.924 **	–0.200
	*B. sinuspersici*	0.957 **	0.982 **	–0.400
***H. bacciferum***	*E. verticillata*	0.905 **	0.802 **	1.000 **
	*S. imbricata*	–0.213	0.067	–0.200
	*B. sinuspersici*	–0.157	–0.085	–0.400
***E. verticillata***	*S. imbricata*	–0.049	0.152	–0.200
	*B. sinuspersici*	–0.064	0.088	–0.400
***S. imbricata***	*B. sinuspersici*	0.936 **	0.881 **	0.800

** Correlation is significant at the 0.01 level (two-tailed).

**Table 5:** Diversity indices of the 23 EF species identified from the six wild stress-tolerant plant hosts.

Plant	*H. salicornicum*	*S.* *kali*	*H. bacciferum*	*E. verticillata*	*S. imbricata*	*B. sinuspersici*	Total
Index							
***No. of samples***	100	100	100	100	100	100	600
***No. of isolates recovered***	72	68	75	75	90	75	455
***Richness (R)***	6	3	5	5	7	6	30
***Shannon’s diversity index (H′)***	0.23	0.17	0.17	0.14	0.1	0.14	0.2
***Evenness (E)***	0.33	0.25	0.25	0.2	0.14	0.2	0.29
***Simpson’s Dominance Index (D′)***	0.8922	0.9144	0.9217	0.9412	0.9524	0.9366	0.9333
***Simpson’s Index of Diversity (1 – D′)***	0.1078	0.0856	0.0783	0.0588	0.0476	0.0634	0.0667
***Berger Parker Dominance Index*** ***p_imax_***	94.4%	95.6%	96.0%	97.0%	97.6%	96.7%	96.6%

## Data Availability

The following information was supplied regarding data availability: Raw data are available as Supplemental Files.

## Supplementary Materials

The following supporting information can be downloaded at: https://www.mdpi.com/article/10.3390/jof8030241/s1, Figure S1: Mean soil physico-chemical characteristics (*n* = 10) of the study area: (A). Soil particle composition and percentage of organic matter (OM%) and organic carbon (OC%), and (B). Macro- and micro-nutrient concentrations in ppm and cation-exchange capacity (CEC) value in cmol(+)/kg of the soil; Figure S2: Average climatic characteristics of the study area: (A). Monthly and annual averages of the lowest and highest temperatures in °C, and (B). Monthly and annual averages of the relative hu-midity (RH%) and rainfall in mm; Figure S3: Representative culture growths and spore morphology of the identified endophytic fungi. (A). Fungal growths, and (B). Fungal sporulation; Figure S4: The isolation rate of EF in the 6 stress-tolerant plant hosts. The lower case letters on the columns refer to significance level. Different letters refer to significant difference and the same letters refer to insignificant difference at *p* < 0.05 using LSD post hoc tests; Figure S5: The percentage colonization frequency (CF%) of EF in the 6 stress-tolerant plant hosts. The lower case letters on the columns refer to significance level. Different letters refer to significant difference and the same letters refer to insignificant difference at *p* < 0.05 using LSD post hoc tests; Table S1: Accession numbers of the endophytic fungal species identified in the present study and those of the closest species on Genbank.

## References

[1] Aly A.H., Debbab A., Proksch P. (2011). Fungal endophytes: Unique plant inhabitants with great promises. Appl. Microbiol. Biotechnol..

[2] Dias A.C., Costa F.E., Andreote F.D., Lacava P.T., Teixeira M.A., Assumpção L.C., Araújo W.L., Azevedo J.L., Melo I.S. (2009). Isolation of micropropagated strawberry endophytic bacteria and assessment of their potential for plant growth promotion. World J. Microbiol. Biotechnol..

[3] Lucero M., Barrow J.R., Osuna P., Reyes I. (2008). A cryptic microbial community persists within micropropagated *Bouteloua eriopoda* (Torr.) Torr. cultures. Plant Sci..

[4] Krings M., Taylor T.N., Hass H., Kerp H., Dotzler N., Hermsen E.J. (2007). Fungal endophytes in a 400-million-yr-old land plant: Infection pathways, spatial distribution, and host responses. New Phytol..

[5] Hawksworth D.C., Rossman A.Y. (1987). Where are the undescribed fungi?. Phytopathology.

[6] Deshmukh S., Hückelhoven R., Schäfer P., Imani J., Sharma M., Weiss M., Waller F., Kogel K.-H. (2006). The root endophytic fungus *Piriformospora indica* requires host cell death for proliferation during mutualistic symbiosis with barley. Proc. Natl. Acad. Sci. USA.

[7] Gutierrez R.M., Gonzalez A.M., Ramirez A.M. (2012). Compounds derived from endophytes: A Review of Phytochemistry and Pharmacology. Curr. Med. Chem..

[8] Debbab A., Aly A.H., Edrada-Ebel R.A., Müller W.E., Mosaddak M., Hakiki A., Ebel R., Proksch P. (2009). Bioactive secondary metabolites from the endophytic fungus *Chaetomium* sp. isolated from *Salvia officinalis* growing in Morocco. Biotechnol. Agron. Soc. Environ..

[9] Banožić M., Jokić S., Ačkar Đ., Blažić M., Šubarić D. (2020). Carbohydrates—Key Players in Tobacco Aroma Formation and Quality Determination. Molecules.

[10] Zhang H.W., Song Y.C., Tan R.X. (2006). Biology and chemistry of endophytes. Nat. Prod. Rep..

[11] Huang W.Y., Cai Y.Z., Surveswaran S., Hyde K.D., Corke H., Sun M. (2009). Molecular phylogenetic identification of endophytic fungi isolated from three *Artemisia* species. Fungal Divers..

[12] Barge E.G., Leopold D.R., Peay K.G., Newcombe G., Busby P.E. (2019). Differentiating spatial from environmental effects on foliar fungal communities of *Populus trichocarpa*. J. Biogeogr..

[13] Liu J., Zhang X., Wang H., Hui X., Wang Z., Qiu W. (2018). Long-term nitrogen fertilization impacts soil fungal and bacterial community structures in a dry-land soil of Loess Plateau in China. J. Soils Sediments.

[14] Carroll G. (1988). Fungal endophytes in stems and leaves: From latent pathogen to mutualistic symbiont. Ecology.

[15] Jia M., Chen L., Xin H.-L., Zheng C.-J., Rahman K., Han T., Qin L.-P. (2016). A friendly relationship between endophytic fungi and medicinal plants: A systematic review. Front. Microbiol..

[16] Hawksworth D.L., Lücking R. (2017). Fungal diversity revisited: 2.2 to 3.8 million species. Microbiol. Spectr..

[17] Petrini O., Andrews J.H., Hirano S.S. (1991). Fungal endophytes of tree leaves. Microbial Ecology of Leaves.

[18] Sridhar K., Raviraja N. (1995). Endophytes. A crucial issue. Curr. Sci..

[19] Hatamzadeh S., Rahnama K., Nasrollahnejad S., Fotouhifar K.B., Hemmati K., White J.F., Taliei F. (2020). Isolation and identification of L-asparaginase-producing endophytic fungi from the Asteraceae family plant species of Iran. PeerJ.

[20] Karlinski L., Rudawska M., Kieliszewska-Rokicka B., Leski T. (2010). Relationship between genotype and soil environment during colonization of poplar roots by mycorrhizal and endophytic fungi. Mycorrhiza.

[21] Stegen J.C., Lin X., Konopka A.E., Fredrickson J.K. (2012). Stochastic and deterministic assembly processes in subsurface microbial communities. ISME J..

[22] Edwards J., Johnson C., Santos-Medellín C., Lurie E., Sundaresan V. (2015). Structure, variation, and assembly of the root-associated microbiomes of rice. Proc. Natl. Acad. Sci. USA.

[23] Nuccio E.E., Anderson-Furgeson K.Y., Estera J., Pett-Ridge P., De Valpine E.L., Brodie M.K., Firestone J. (2016). Climate and edaphic controllers influence rhizosphere community assembly for a wild annual grass. Ecology.

[24] Yang H., Ye W., Ma J., Zeng D., Rong Z., Xu M., Wang Y., Zheng X. (2018). Endophytic fungal communities associated with field-grown soybean roots and seeds in the Huang-Huai region of China. PeerJ.

[25] Bamisile B.S., Dash C.K., Akutse K.S., Keppanan R., Wang L. (2018). Fungal Endophytes: Beyond Herbivore Management. Front. Microbiol..

[26] Hardoim P.R., van Overbeek L.S., Berg G., Pirttilä A.M., Compant S., Campisano A., Döring M., Sessitsch A. (2015). The Hidden World within Plants: Ecological and evolutionary considerations for defining functioning of microbial endophytes. Microbiol. Mol. Biol. Rev..

[27] Huang Q., An H., Song H., Mao H., Shen W., Dong J. (2015). Diversity and biotransformative potential of endophytic fungi associated with the medicinal plant *Kadsura angustifolia*. Res. Microbiol..

[28] Deepthi V.C., Sumathi S., Faisal M., Elyas K.K. (2018). Isolation and identification of endophytic fungi with antimicrobial activities from the leaves of *Elaeocarpus sphaericus* (gaertn.) K. Schum. and *Myristica fragrans* houtt. Int. J. Pharm. Sci. Res..

[29] Chauhan N.M., Gutama A.D., Aysa A. (2019). Endophytic fungal diversity isolated from different agro-ecosystem of Enset (*Ensete ventericosum*) in Gedeo zone, SNNPRS, Ethiopia. BMC Microbiol..

[30] Su-Han N.H., Songkumarn P., Nuankaew S., Boonyuen N., Piasai O. (2019). Diversity of sporulating rice endophytic fungi associated with Thai rice cultivars (*Oryza sativa* L.) cultivated in Suphanburi and Chainat Provinces, Thailand. Curr. Res. Environ. Appl. Mycol. (J. Fungal Biol.).

[31] Tejesvi M.V., Kajula M., Mattila S., Pirttilä A.M. (2011). Bioactivity and genetic of endophytic fungi in *Rhododendron tomentosun* Harmaja. Fungal Divers..

[32] Dang H., Zhang T., Wang Z., Li G., Zhao W., Lv X., Zhuang L. (2021). Differences in the endophytic fungal community and effective ingredients in root of three *Glycyrrhiza* species in Xinjiang, China. PeerJ.

[33] Wang Y.A., Zeng Q.G., Zhang Z.B., Yan R.M., Wang L.Y., Zhu D. (2011). Isolation and characterization of endophytic huperzine A-producing fungi from *Huperzia serrata*. J. Ind. Microbiol. Biotechnol..

[34] Gull A., Lone A.A., Wani N.U.I., de Oliveira A.B. (2019). Biotic and abiotic stresses in plants. Abiotic and Biotic Stress in Plants.

[35] Chaudhary S.A. (1999). Flora of the Kingdom of Saudi Arabia Illustrated Series.

[36] Schulz B., Wanke U., Draeger S., Aust H.-J. (1993). Endophytes from herbaceous plants and shrubs: Effectiveness of surface sterilization methods. Mycol. Res..

[37] Arx J.A. (1981). The Genera of Fungi Sporulating in Pure Culture.

[38] Ellis M.B. (1971). Dematiaceous Hyphomycetes.

[39] Ellis M.B. (1976). More Dematiaceous Hyphomycetes.

[40] Kirk P., Cannon P., Minter D., Stalpers J. (2008). Dictionary of the Fungi.

[41] Seifert K., Morgan-Jones G., Gams W., Kendrick B. (2011). The Genera of Hyphomycetes.

[42] Sutton B.C. (1980). The Coelomycetes.

[43] White T.J., Bruns T., Lee S., Taylor J., Innis M.A., Gelfand D.H., Sninsky J.J., White T.J. (1990). Amplification and direct sequencing of fungal ribosomal RNA genes for phylogenetics. PCR Protocols: A Guide to Methods and Applications.

[44] Hata K., Futai K. (1995). Endophytic fungi associated with healthy pine needles and needles infested by the pine needle gall midge, *Thecodiplosis japonensis*. Can. J. Bot..

[45] Suryanarayanan T.S., Venkatesan G., Murali T.S. (2003). Endophytic fungal communities in leaves of tropical forest trees: Diversity and distribution patterns. Curr. Sci..

[46] Shannon C.E., Wiener W. (1964). The Mathematical Theory of Communication.

[47] Simpson E.H. (1949). Measurement of diversity. Nature.

[48] Berger W.H., Parker F.L. (1970). Diversity of planktonic foraminifera in deep-sea sediments. Science.

[49] Rajapaksha P., Elbourne A., Gangadoo S., Brown R., Cozzolino D., Chapman J. (2019). A review of methods for the detection of pathogenic microorganisms. Analyst.

[50] Rhoads A., Au K.F. (2015). PacBio sequencing and its applications. Genom. Proteom. Bioinf..

[51] Tibpromma S., Hyde K.D., Bhat J.D., Mortimer P.E., Xu J., Promputtha I., Doilom M., Jun-Bo Y., Tang A.M., Karunarathna S.C. (2018). Identification of endophytic fungi from leaves of Pandanaceae based on their fungal taxa and DNA sequence data from southern Thailand. MycoKeys.

[52] Yao H., Sun X., He C., Maitra P., Li X.-C., Guo L.-D. (2019). Phyllosphere epiphytic and endophytic fungal community and network structures differ in a tropical mangrove ecosystem. Microbiome.

[53] Bajinka O., Secka O. (2017). Integration of molecular methods into microbiological diagnostics. App. Microbiol. Open Access.

[54] Rosselló-Mora R., Kampfer P., Bull A.T. (2004). Defining microbial diversity-the species concept for prokaryotic and eukaryotic microorganisms. Microbial Diversity and Bioprospecting.

[55] Edgar R. (2018). Accuracy of taxonomy prediction for 16S rRNA and fungal ITS sequences. PeerJ.

[56] Edgar R. (2018). Updating the 97% identity threshold for 16S ribosomal RNA OTUs. Bioinformatics.

[57] Vu D., Groenewald M., de Vries M., Gehrmann T., Stielow B., Eberhardt U., Al-Hatmi A., Groenewald J.Z., Cardinali G., Houbraken J. (2019). Large-scale generation and analysis of filamentous fungal DNA barcodes boosts coverage for kingdom fungi and reveals thresholds for fungal species and higher taxon delimitation. Stud. Mycol..

[58] Pearson W. (2013). An introduction to sequence similarity (“Homology”) searching. Curr. Protoc. Bioinform..

[59] Castellanos-González L., de Mello-Prado R., Silva-Campos C.N., Barbosa-da S., Júnior G. (2020). Development of the southern corn leaf blight caused by *Bipolaris maydis* (teleomorph: *Cochliobolus*
*heterostrophus* in sweet corn as a function of nitrogen, potassium, and silicon under greenhouse conditions. Cienc. Y Tecnol. Agropecu..

[60] Lamprecht S.C., Tewoldemedhin Y.T., Botha W.J., Calitz F.J. (2011). *Fusarium graminearum* species complex associated with maize crowns and roots in the KwaZulu-Natal Province of South Africa. Plant Dis..

[61] Pennerman K.K., Yin G., Glenn A.E., Bennett J.W. (2020). Identifying candidate *Aspergillus* pathogenicity factors by annotation frequency. BMC Microbiol..

[62] Sajad A.M., Abid J.H. (2017). Fungi associated with the spoilage of post-harvest tomato fruits and their frequency of occurrences in different markets of Jabalpur, Madhya-Pradesh, India. Int. J. Curr. Res. Rev..

[63] Qiang X., Ding J., Lin W., Li Q., Xu C., Zheng Q., Li Y. (2019). Alleviation of the detrimental effect of water deficit on wheat (*Triticum aestivum* L.) growth by an indole acetic acid-producing endophytic fungus. Plant Soil.

[64] Ali R., Gul H., Hamayun M., Rauf M., Iqbal A., Shah M., Hussain A., Bibi H., Lee I.-J. (2021). *Aspergillus awamori* ameliorates the physicochemical characteristics and mineral profile of mung bean under salt stress. Chem. Biol. Technol. Agric..

[65] Hamayun I.M., Hussain A., Iqbal A., Khan S.A., Lee I.-J. (2020). *Aspergillus niger* boosted heat stress tolerance in sunflower and soybean via regulating their metabolic and antioxidant system. J. Plant Interact..

[66] Lubna L., Khan M.A., Asaf S., Jan R., Waqas M., Kim K., Lee I.-J. (2020). Plant growth promoting *Bipolaris* sp. CSL-1 mitigate salinity stress in soybean via altering endogenous phytohormonal level, antioxidants and genes expression. Res. Sq..

[67] Sampangi-Ramaiah M.H., Ravishankar K.V., Nataraja K.N., Shaanker R.U. (2019). Endophytic fungus, *Fusarium* sp. reduces alternative splicing events in rice plants under salinity stress. Plant Physiol. Rep..

[68] Kavroulakis N., Doupis G., Papadakis I.E., Ehaliotis C., Papadopoulou K.K. (2018). Tolerance of tomato plants to water stress is improved by the root endophyte *Fusarium solani* FsK. Rhizosphere.

[69] deLamo F.J., Takken F.L. (2020). Biocontrol by *Fusarium oxysporum* using endophyte mediated resistance. Front. Plant Sci..

[70] delaCruz T.E., Wagner S., Schulz B. (2006). Physiological responses of marine *Dendryphiella* species from different geographical locations. Mycol. Prog..

[71] d’Errico G., Aloj V., Flematti G.R., Sivasithamparam K., Worth C.M., Lombardi N., Ritieni A., Marra R., Lorito M., Vinale F. (2021). Metabolites of a *Drechslera* sp. endophyte with potential as biocontrol and bioremediation agent. Nat. Prod. Res..

[72] Kaaniche F., Hamed A., Abdel-Razek A.S., Wibberg D., Abdissa N., El Euch I.Z., Allouche N., Mellouli L., Shaaban M., Sewald N. (2019). Bioactive secondary metabolites from new endophytic fungus *Curvularia*. sp isolated from *Rauwolfia macrophylla*. PLoS ONE.

[73] Leitão A.L., Enguita F.J. (2016). Gibberellins in *Penicillium* strains: Challenges for endophyte-plant host interactions under salinity stress. Microbiol. Res..

[74] Mou L.Y., Xin X.L., Chen L., Dong P.P., Lan R., Su D.H., Huang J., Wang J.H., Zhan L.B. (2014). Biotransformation of resibufogenin by *Actinomucor elegans*. J. Asian Nat. Prod. Res..

[75] Valli P.S., Muthukumar T. (2018). Dark septate root endophytic fungus *Nectria haematococca* improves tomato growth under water limiting conditions. Indian J. Microbiol..

[76] Xiong D.-S., Yang Y.-B., Hu B.-Y., Miao C.-P., Wang Y.-L., Zou J.-M., Li L., Li Y.-Q., Luo X.-D., Zhao L.-X. (2021). Myrothins A-F from endophytic fungus *Myrothecium* sp. BS-31 harbored in *Panax notoginseng*. Chem. Biodivers..

[77] Zimmerman N.B., Vitousek P.M. (2012). Fungal endophyte communities reflect environmental structuring across a Hawaiian landscape. Proc. Natl. Acad. Sci. USA.

[78] Liu D., Cai J., He H., Yang S., Chater C.C.C., Yu F. (2022). Anemochore Seeds Harbor Distinct Fungal and Bacterial Abundance, Composition, and Functional Profiles. J. Fungi.

[79] Rodriguez R.J., Henson J., Van Volkenburgh E., Hoy M., Wright L., Beckwith F., Kim Y., Redman R.S. (2008). Stress tolerance in plants via habitat-adapted symbiosis. ISME J..

[80] Aletaha R., Safari S.A., Zafari D. (2018). A survey on the endophytic fungi in the roots of Chenopodiaceae under different environmental conditions. Mycosphere.

[81] Rajagopal S., Kumar R.A., Deevi D.S., Sathyanarayana C., Rajagopalan R. (2010). A potential cancer therapeutic agent isolated from *Andrographis paniculata*. J. Exp. Ther. Oncol..

[82] Zhou Z., Zhang C., Zhou W., Li W., Chu L., Yan J., Li H. (2014). Diversity and plant growth-promoting ability of endophytic fungi from the five flower plant species collected from Yunnan, Southwest China. J. Plant Interact..

[83] Khalmuratova I., Choi D.-H., Jeong M.-J., Oh Y., Kim Y.-G., Lee I.-J., Choo Y.-S., Kim J.-G. (2020). Diversity and Plant Growth-Promoting Effects of Fungal Endophytes Isolated from Salt-Tolerant Plants. J. Microbiol. Biotechnol..

[84] Khalmuratova I., Choi D.-H., Yoon H.-J., Yoon T.-M., Kim J.-G. (2021). Diversity and Plant Growth Promotion of Fungal Endophytes in Five Halophytes from the Buan Salt Marsh. J. Microbiol. Biotechnol..

[85] Baron N.C., Rigobelo E.C. (2022). Endophytic fungi: A tool for plant growth promotion and sustainable agriculture. Mycology.

[86] Hodkinson T., Doohan F., Saunders M., Murphy B., Hodkinson T., Doohan F., Saunders M., Murphy B. (2019). Role of endophytes in growth and biotic and abiotic stress resistance. Endophytes for a Growing World.

[87] Rho H., Hsieh M., Kandel S.L., Cantillo J., Doty S.L., Kim S.-H. (2018). Do endophytes promote growth of host plants under stress? A meta-analysis on plant stress mitigation by endophyte *Hyungmin*. Microb. Ecol..

[88] Almario J., Jeena G., Wunder J., Langen G., Zuccaro A., Coupland G., Bucher M. (2017). Root-associated fungal microbiota of nonmycorrhizal *Arabis alpina* and its contribution to plant phosphorus nutrition. Proc. Natl. Acad. Sci. USA.

