# Supplementary material for: Composition and Diversity of the Culturable Endophytic Community of Six Stress-Tolerant Dessert Plants Grown in Stressful Soil in a Hot Dry Desert Region
Source: J Fungi (Basel). 2022 Feb 28;8(3):241. doi: 10.3390/jof8030241 (PMC8948987; doi:10.3390/jof8030241)
Supplement: Supplementary file 1 [file jof-08-00241-s001.zip › Supplementary-R2/Table S1.pdf]

Table S1. Accession numbers of the endophytic fungal species identified in the present study and those of the closest species on Genbank.

Organism name	Accession number of the species	Accession number of the closest species on Genbank
Alternaria infectoria	*MZ636612*	MK828116.1
Penicillium citrinum	*MZ636613*	MG736724.1
Aspergillus terreus	*MZ636614*	MF503667.1
Bipolaris spicifera	*MZ636615*	MT649586.1
Fusarium venenatum	*MZ636616*	MT649588.1
Aspergillus flavus	*MZ636617*	MN453362.1
Myrothecium sp.	*MZ636618*	MT649553.1
Alternaria alternata	*MZ636619*	MK500615.1
Nectria cinnabarina	*MZ636620*	AB237663.1
Aspergillus niger	*MZ636621*	MN535797.1
Alternaria botrytis	*MZ636622*	MZ314140.1
Fusarium oxysporum	*MZ636623*	LT841236.1
Curvularia lunata	*MZ636624*	MH102208.1
Aspergillus clavatus	*MZ636625*	AB002070.1
Fusarium proliferatum	*MZ636626*	MT445785.1
Drechslera sp.	*MZ636627*	KU981163.2
Fusarium fujikuroi	*MZ636628*	LS422781.1
Dendryphiella salina	*MZ636629*	EU848586.1
Actinomucor elegans	*MZ636630*	MN995506.1
Bipolaris sorokiniana	*MZ636631*	MH014993.1
Penicillium griseofulvum	*MZ636632*	MG736723.1
Fusarium solani	*MZ636633*	MK027282.1
Aspergillus awamori	*MZ636634*	MK530131.1
