# Supplementary figures and images for: Composition and Diversity of the Culturable Endophytic Community of Six Stress-Tolerant Dessert Plants Grown in Stressful Soil in a Hot Dry Desert Region
Source: J Fungi (Basel). 2022 Feb 28;8(3):241. doi: 10.3390/jof8030241 (PMC8948987; doi:10.3390/jof8030241)

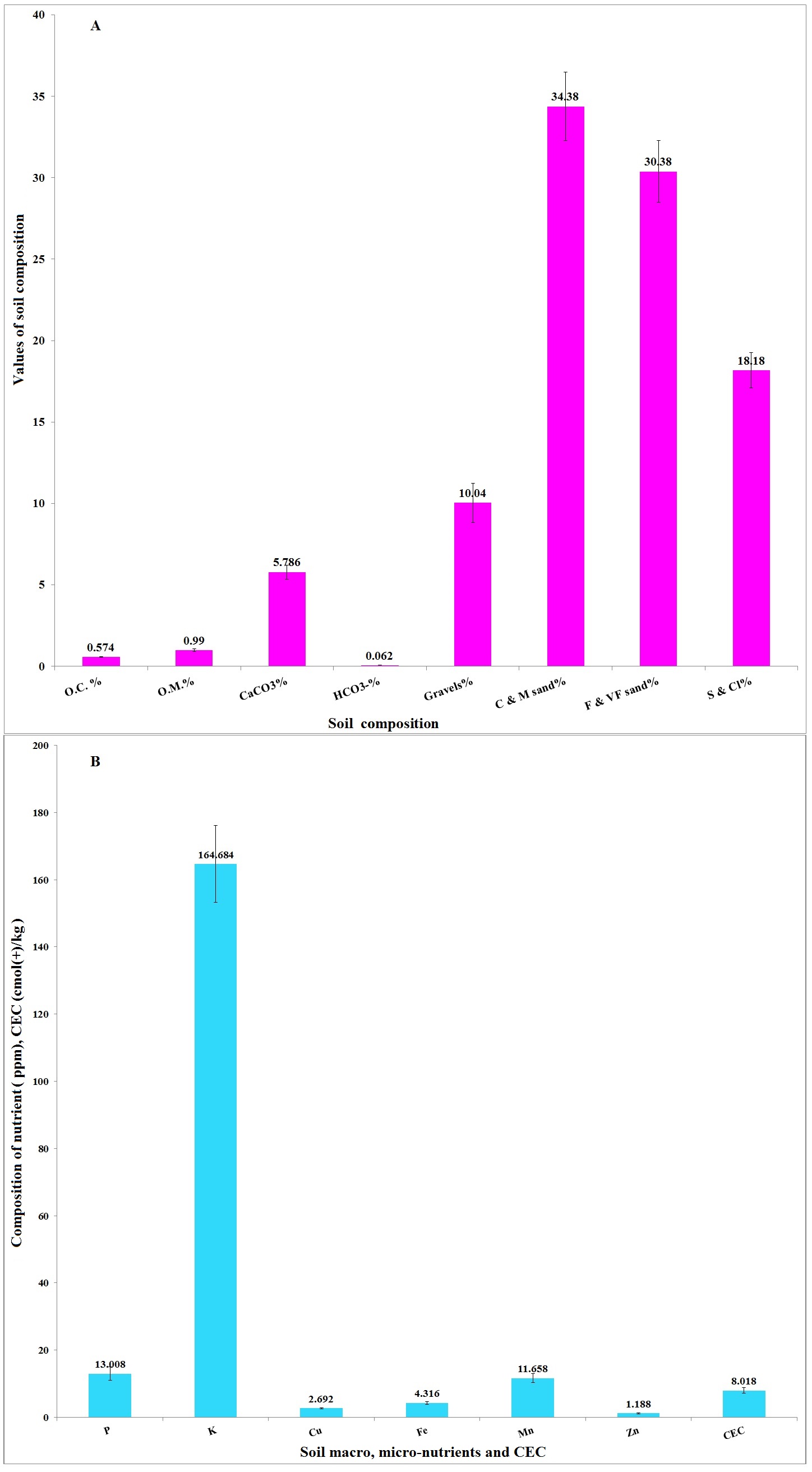

Supplement: Supplementary file 1 [file jof-08-00241-s001.zip › Supplementary-R2/Figure. S1- Soil Characteristics.jpg]

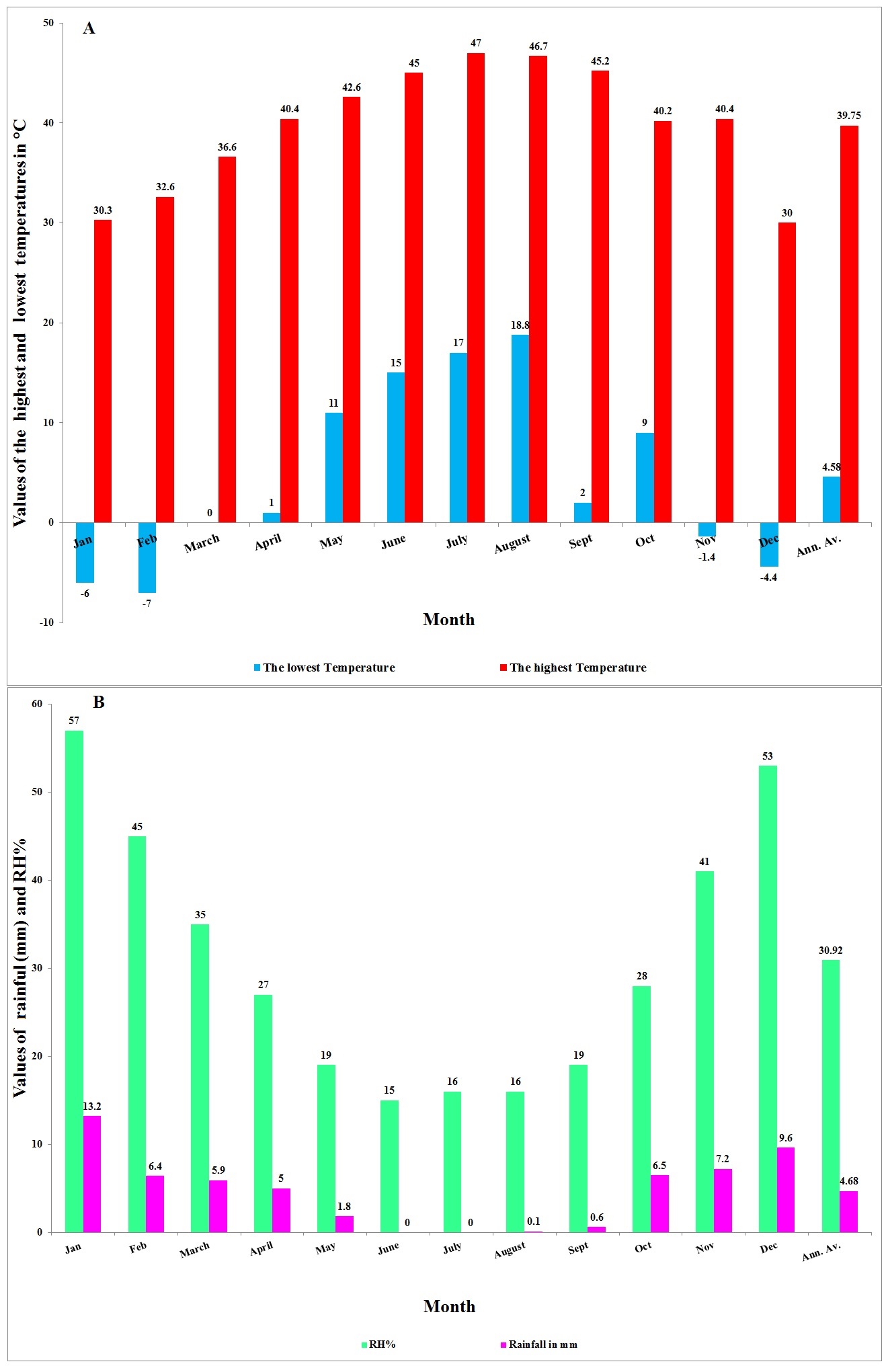

Supplement: Supplementary file 1 [file jof-08-00241-s001.zip › Supplementary-R2/Figure. S2- Climate.jpg]

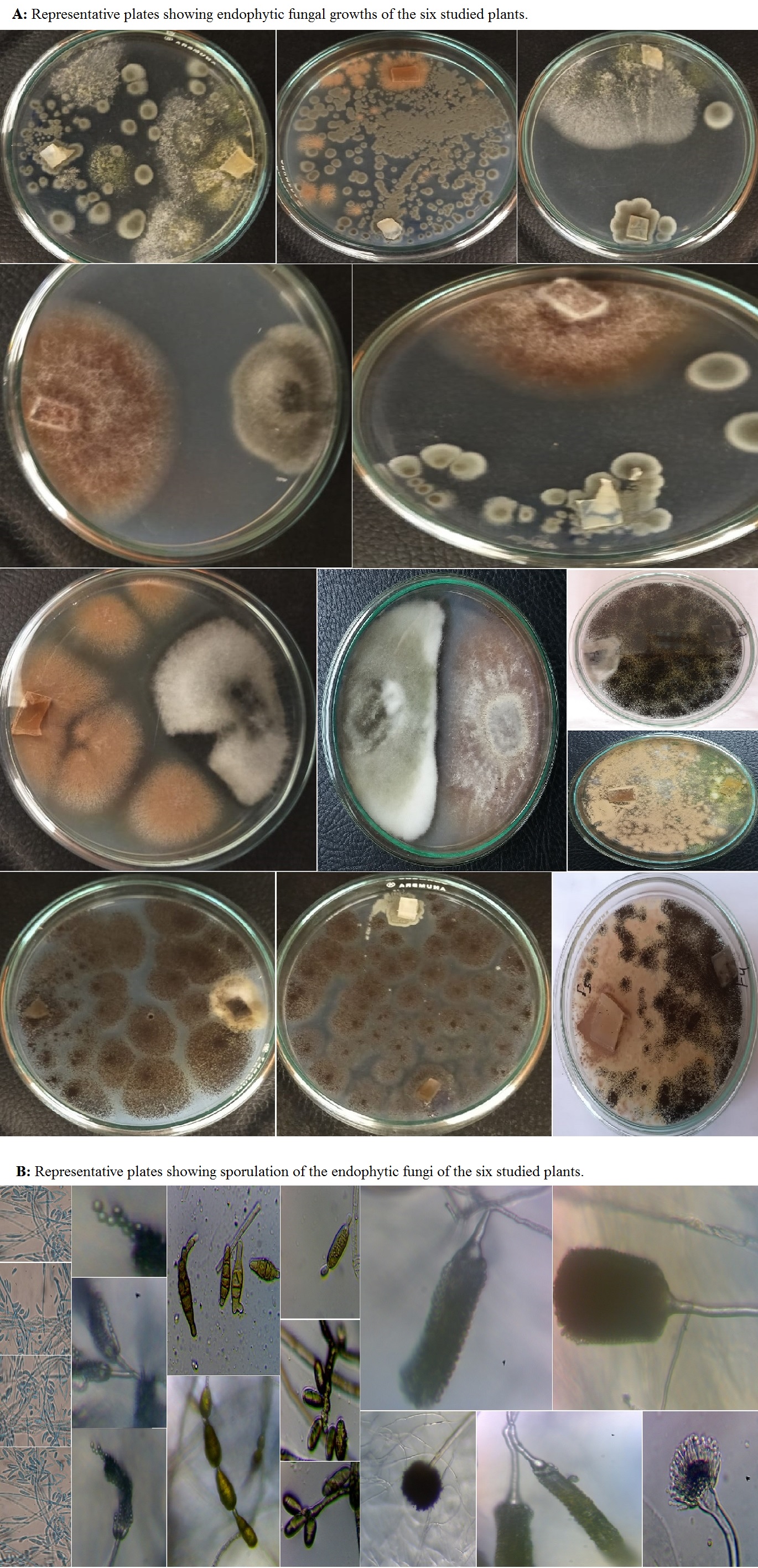

Supplement: Supplementary file 1 [file jof-08-00241-s001.zip › Supplementary-R2/Figure. S3-Plates of EF.jpg]

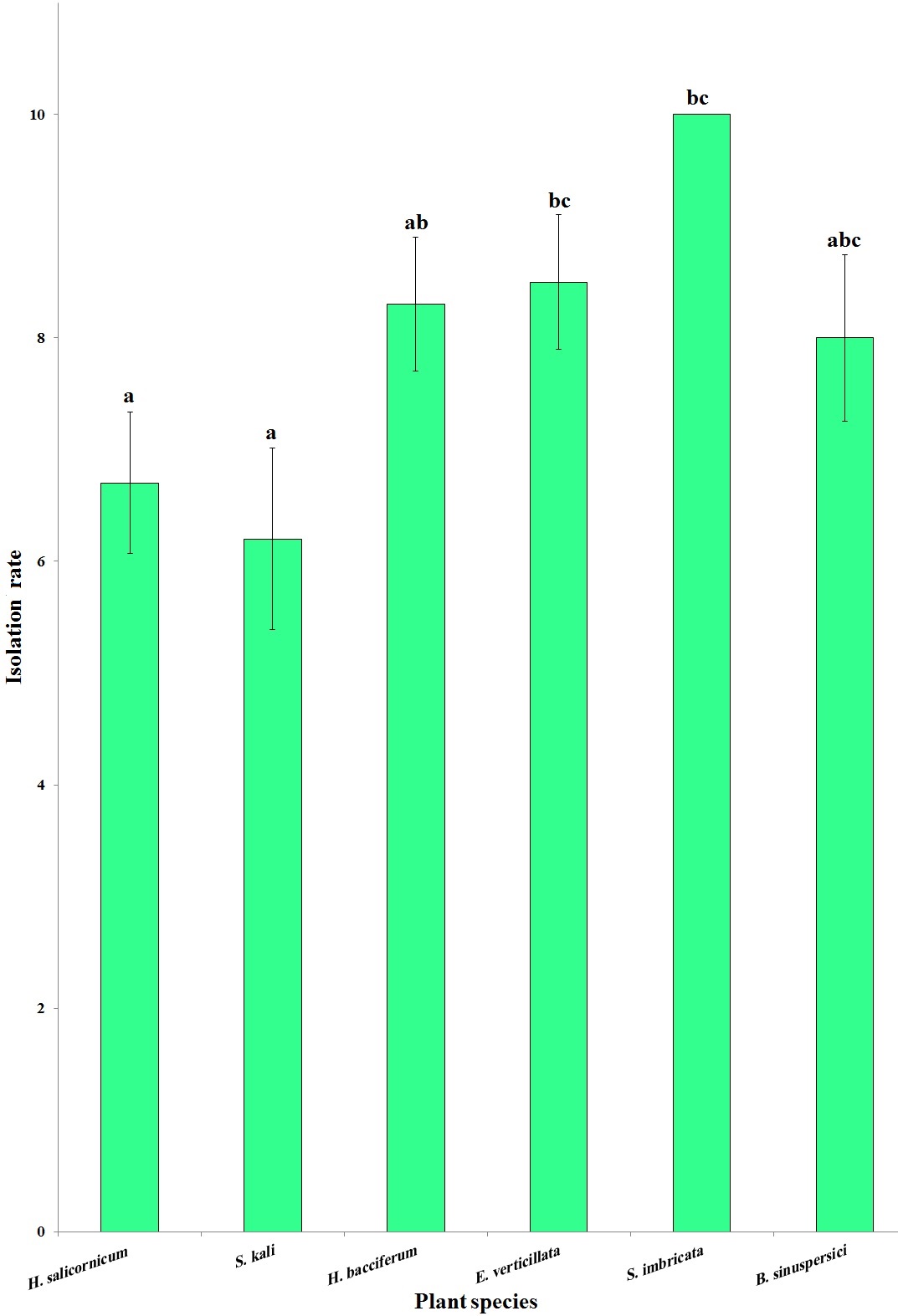

Supplement: Supplementary file 1 [file jof-08-00241-s001.zip › Supplementary-R2/Figure. S4-Isolation Rate.jpg]

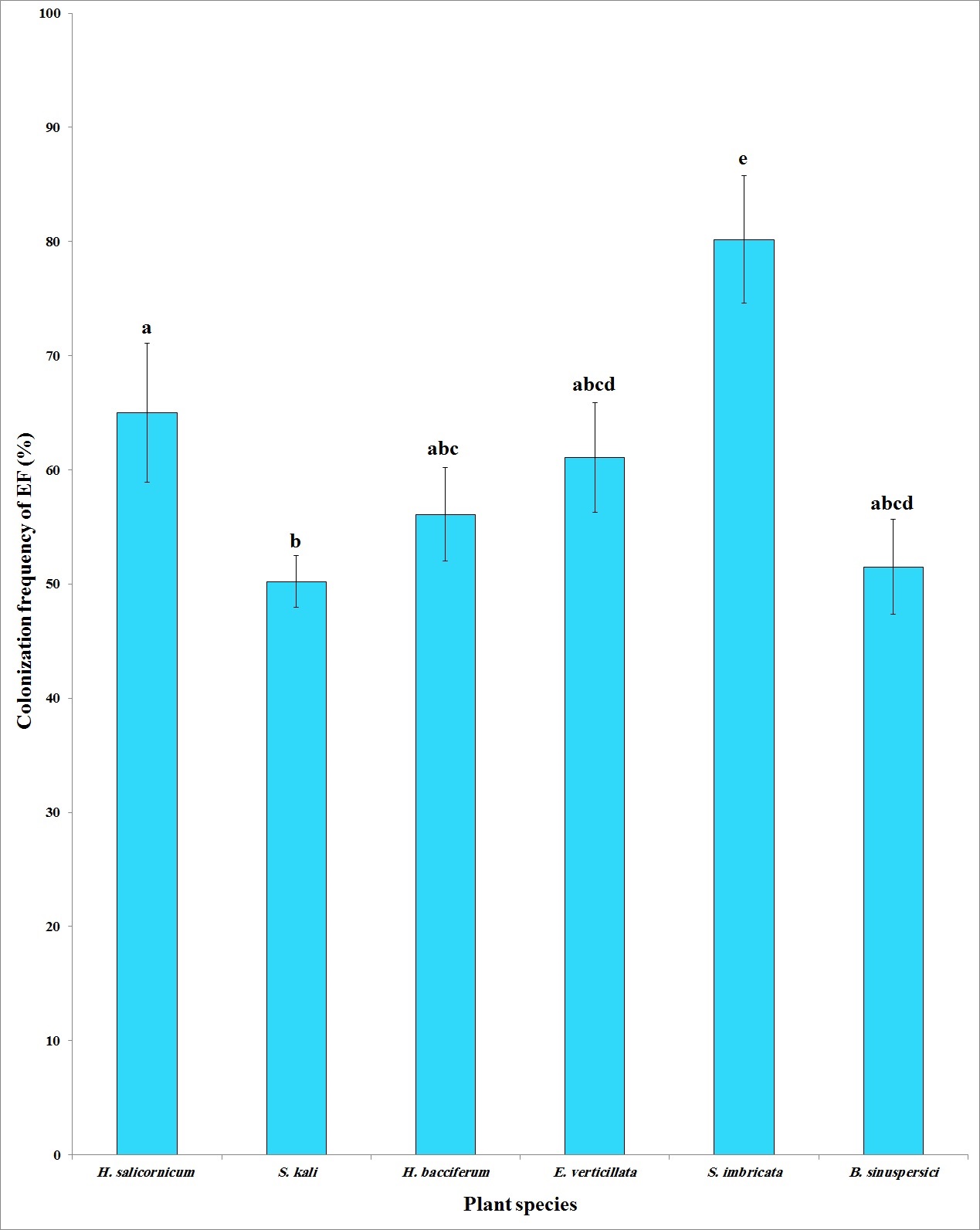

Supplement: Supplementary file 1 [file jof-08-00241-s001.zip › Supplementary-R2/Figure. S5-CF%.jpg]
